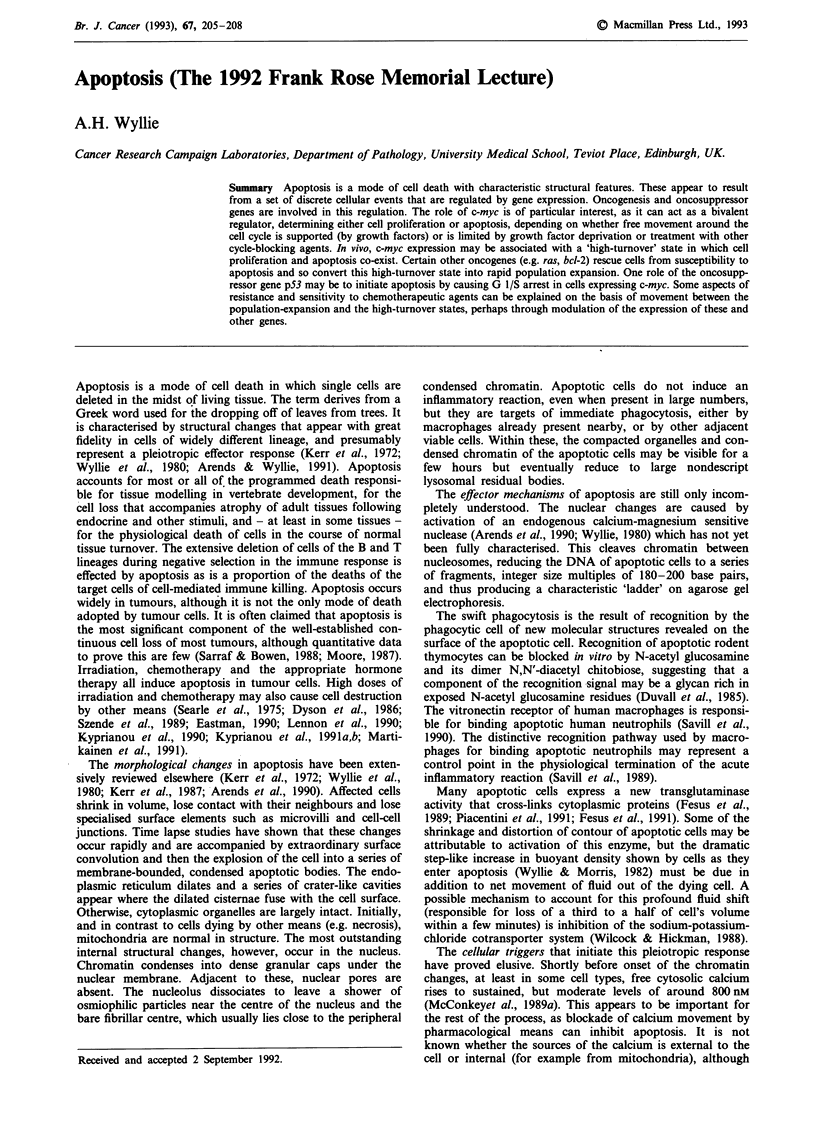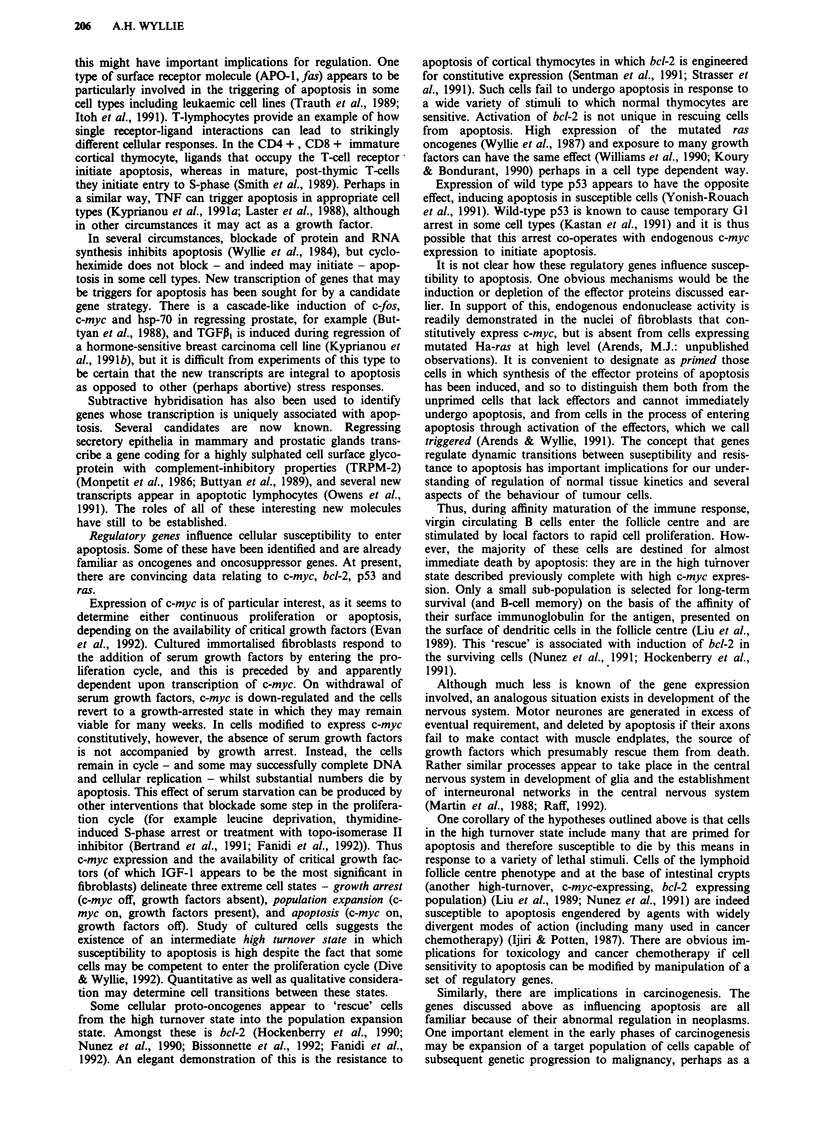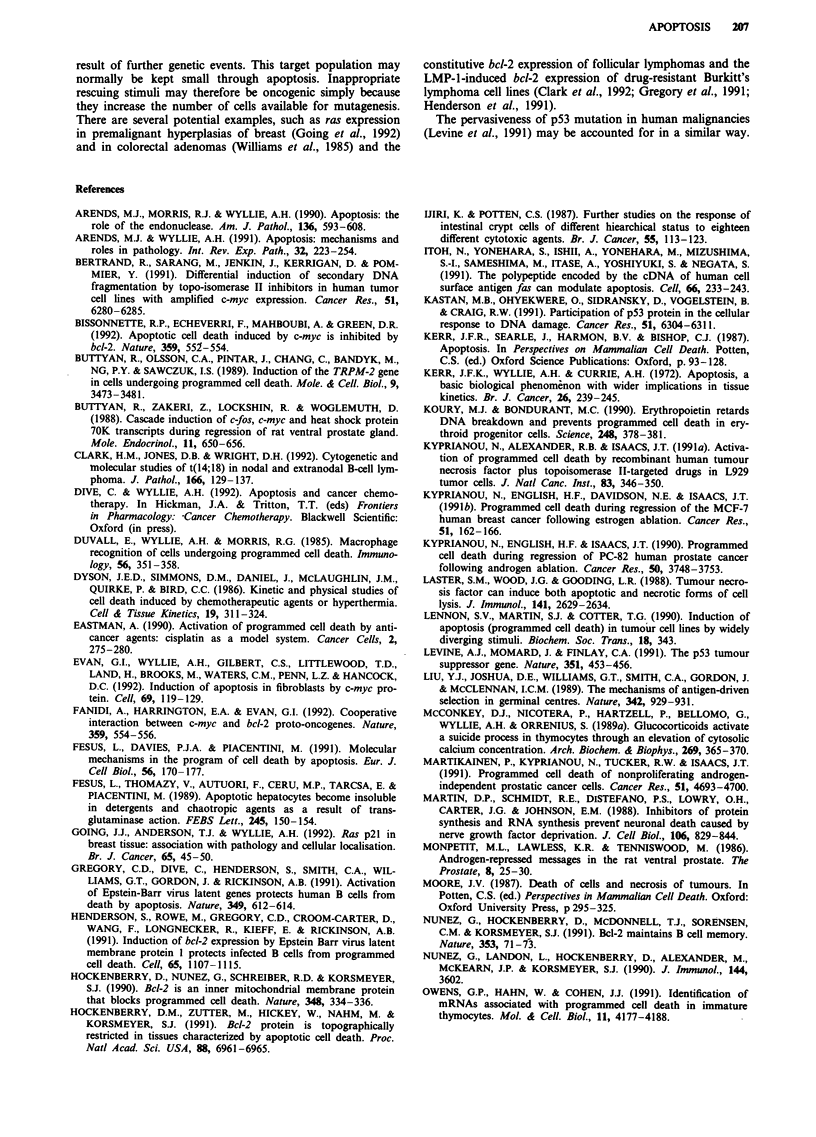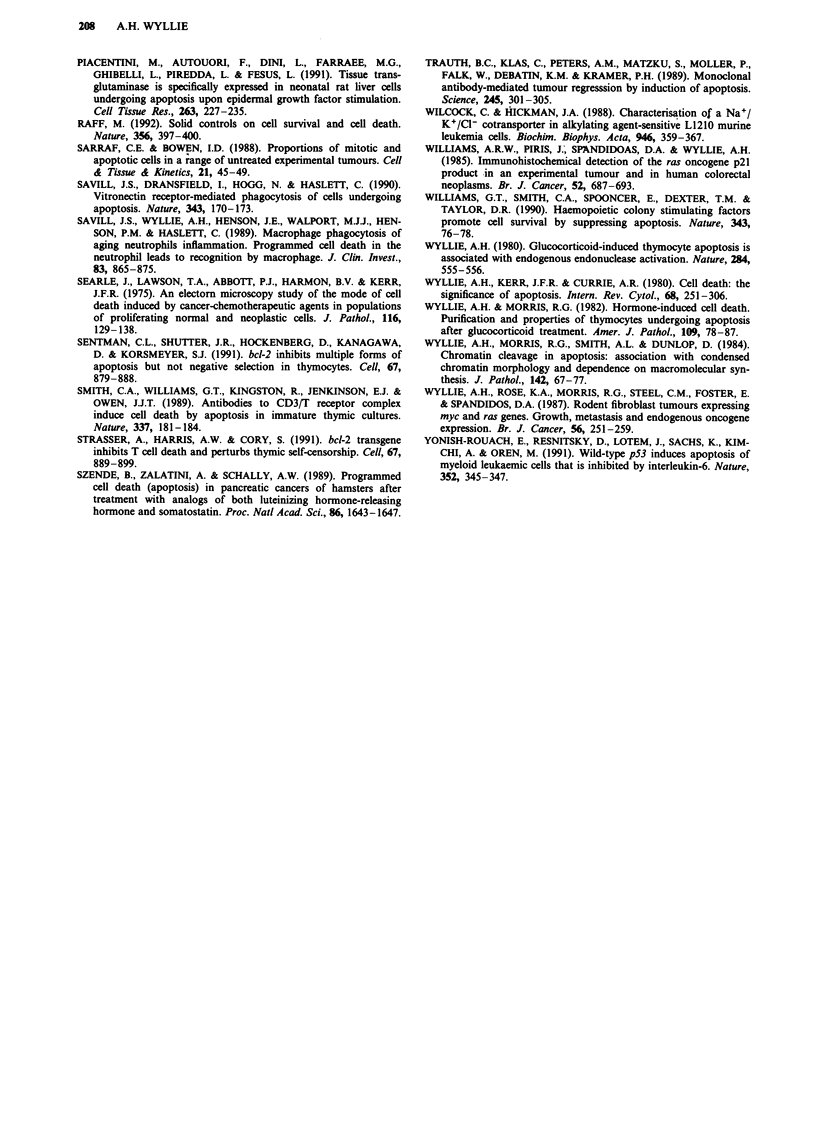# Apoptosis (the 1992 Frank Rose Memorial Lecture).

**DOI:** 10.1038/bjc.1993.40

**Published:** 1993-02

**Authors:** A. H. Wyllie

**Affiliations:** Department of Pathology, University Medical School, Edinburgh, UK.

## Abstract

Apoptosis is a mode of cell death with characteristic structural features. These appear to result from a set of discrete cellular events that are regulated by gene expression. Oncogenesis and oncosuppressor genes are involved in this regulation. The role of c-myc is of particular interest, as it can act as a bivalent regulator, determining either cell proliferation or apoptosis, depending on whether free movement around the cell cycle is supported (by growth factors) or is limited by growth factor deprivation or treatment with other cycle-blocking agents. In vivo, c-myc expression may be associated with a 'high-turnover' state in which cell proliferation and apoptosis co-exist. Certain other oncogenes (e.g. ras, bcl-2) rescue cells from susceptibility to apoptosis and so convert this high-turnover state into rapid population expansion. One role of the oncosuppressor gene p53 may be to initiate apoptosis by causing G 1/S arrest in cells expressing c-myc. Some aspects of resistance and sensitivity to chemotherapeutic agents can be explained on the basis of movement between the population-expansion and the high-turnover states, perhaps through modulation of the expression of these and other genes.


### Apoptosis (The 1992 Frank Rose Memorial Lecture)

A.H. Wyllie

*Cancer Research Campaign Laboratories, Department of Pathology, University Medical School, Teviot Place, Edinburgh, UK.*

**Summary** Apoptosis is a mode of cell death with characteristic structural features. These appear to result from a set of discrete cellular events that are regulated by gene expression. Oncogenesis and oncosuppressor genes are involved in this regulation. The role of c-*myc* is of particular interest, as it can act as a bivalent regulator, determining either cell proliferation or apoptosis, depending on whether free movement around the cell cycle is supported (by growth factors) or is limited by growth factor deprivation or treatment with other cycle-blocking agents. *In vivo*, c-*myc* expression may be associated with a 'high-turnover' state in which cell proliferation and apoptosis co-exist. Certain other oncogenes (e.g. *ras*, *bcl*-2) rescue cells from susceptibility to apoptosis and so convert this high-turnover state into rapid population expansion. One role of the oncosuppressor gene p*53* may be to initiate apoptosis by causing G 1/S arrest in cells expressing c-*myc*. Some aspects of resistance and sensitivity to chemotherapeutic agents can be explained on the basis of movement between the population-expansion and the high-turnover states, perhaps through modulation of the expression of these and other genes.

Apoptosis is a mode of cell death in which single cells are deleted in the midst of living tissue. The term derives from a Greek word used for the dropping off of leaves from trees. It is characterised by structural changes that appear with great fidelity in cells of widely different lineage, and presumably represent a pleiotropic effector response (Kerr *et al.*, 1972; Wyllie *et al.*, 1980; Arends & Wyllie, 1991). Apoptosis accounts for most or all of the programmed death responsible for tissue modelling in vertebrate development, for the cell loss that accompanies atrophy of adult tissues following endocrine and other stimuli, and – at least in some tissues – for the physiological death of cells in the course of normal tissue turnover. The extensive deletion of cells of the B and T lineages during negative selection in the immune response is effected by apoptosis as is a proportion of the deaths of the target cells of cell-mediated immune killing. Apoptosis occurs widely in tumours, although it is not the only mode of death adopted by tumour cells. It is often claimed that apoptosis is the most significant component of the well-established continuous cell loss of most tumours, although quantitative data to prove this are few (Sarraf & Bowen, 1988; Moore, 1987). Irradiation, chemotherapy and the appropriate hormone therapy all induce apoptosis in tumour cells. High doses of irradiation and chemotherapy may also cause cell destruction by other means (Searle *et al.*, 1975; Dyson *et al.*, 1986; Szende *et al.*, 1989; Eastman, 1990; Lennon *et al.*, 1990; Kyprianou *et al.*, 1990; Kyprianou *et al.*, 1991*a,b*; Martikainen *et al.*, 1991).

The *morphological changes* in apoptosis have been extensively reviewed elsewhere (Kerr *et al.*, 1972; Wyllie *et al.*, 1980; Kerr *et al.*, 1987; Arends *et al.*, 1990). Affected cells shrink in volume, lose contact with their neighbours and lose specialised surface elements such as microvilli and cell-cell junctions. Time lapse studies have shown that these changes occur rapidly and are accompanied by extraordinary surface convolution and then the explosion of the cell into a series of membrane-bounded, condensed apoptotic bodies. The endoplasmic reticulum dilates and a series of crater-like cavities appear where the dilated cisternae fuse with the cell surface. Otherwise, cytoplasmic organelles are largely intact. Initially, and in contrast to cells dying by other means (e.g. necrosis), mitochondria are normal in structure. The most outstanding internal structural changes, however, occur in the nucleus. Chromatin condenses into dense granular caps under the nuclear membrane. Adjacent to these, nuclear pores are absent. The nucleolus dissociates to leave a shower of osmiophilic particles near the centre of the nucleus and the bare fibrillar centre, which usually lies close to the peripheral condensed chromatin. Apoptotic cells do not induce an inflammatory reaction, even when present in large numbers, but they are targets of immediate phagocytosis, either by macrophages already present nearby, or by other adjacent viable cells. Within these, the compacted organelles and condensed chromatin of the apoptotic cells may be visible for a few hours but eventually reduce to large nondescript lysosomal residual bodies.

The *effector mechanisms* of apoptosis are still only incompletely understood. The nuclear changes are caused by activation of an endogenous calcium-magnesium sensitive nuclease (Arends *et al.*, 1990; Wyllie, 1980) which has not yet been fully characterised. This cleaves chromatin between nucleosomes, reducing the DNA of apoptotic cells to a series of fragments, integer size multiples of 180–200 base pairs, and thus producing a characteristic 'ladder' on agarose gel electrophoresis.

The swift phagocytosis is the result of recognition by the phagocytic cell of new molecular structures revealed on the surface of the apoptotic cell. Recognition of apoptotic rodent thymocytes can be blocked *in vitro* by N-acetyl glucosamine and its dimer N,N′-diacetyl chitobiose, suggesting that a component of the recognition signal may be a glycan rich in exposed N-acetyl glucosamine residues (Duvall *et al.*, 1985). The vitronectin receptor of human macrophages is responsible for binding apoptotic human neutrophils (Savill *et al.*, 1990). The distinctive recognition pathway used by macrophages for binding apoptotic neutrophils may represent a control point in the physiological termination of the acute inflammatory reaction (Savill *et al.*, 1989).

Many apoptotic cells express a new transglutaminase activity that cross-links cytoplasmic proteins (Fesus *et al.*, 1989; Piacentini *et al.*, 1991; Fesus *et al.*, 1991). Some of the shrinkage and distortion of contour of apoptotic cells may be attributable to activation of this enzyme, but the dramatic step-like increase in buoyant density shown by cells as they enter apoptosis (Wyllie & Morris, 1982) must be due in addition to net movement of fluid out of the dying cell. A possible mechanism to account for this profound fluid shift (responsible for loss of a third to a half of cell's volume within a few minutes) is inhibition of the sodium-potassium-chloride cotransporter system (Wilcock & Hickman, 1988).

The *cellular triggers* that initiate this pleiotropic response have proved elusive. Shortly before onset of the chromatin changes, at least in some cell types, free cytosolic calcium rises to sustained, but moderate levels of around 800 nM (McConkeyet *al.*, 1989*a*). This appears to be important for the rest of the process, as blockade of calcium movement by pharmacological means can inhibit apoptosis. It is not known whether the sources of the calcium is external to the cell or internal (for example from mitochondria), although

Received and accepted 2 September 1992.

this might have important implications for regulation. One type of surface receptor molecule (APO-1, *fas*) appears to be particularly involved in the triggering of apoptosis in some cell types including leukaemic cell lines (Trauth *et al.*, 1989; Itoh *et al.*, 1991). T-lymphocytes provide an example of how single receptor-ligand interactions can lead to strikingly different cellular responses. In the CD4 +, CD8 + immature cortical thymocyte, ligands that occupy the T-cell receptor initiate apoptosis, whereas in mature, post-thymic T-cells they initiate entry to S-phase (Smith *et al.*, 1989). Perhaps in a similar way, TNF can trigger apoptosis in appropriate cell types (Kyprianou *et al.*, 1991*a*; Laster *et al.*, 1988), although in other circumstances it may act as a growth factor.

In several circumstances, blockade of protein and RNA synthesis inhibits apoptosis (Wyllie *et al.*, 1984), but cycloheximide does not block – and indeed may initiate – apoptosis in some cell types. New transcription of genes that may be triggers for apoptosis has been sought for by a candidate gene strategy. There is a cascade-like induction of c-*fos*, c-*myc* and hsp-70 in regressing prostate, for example (Buttyan *et al.*, 1988), and TGF$\beta_1$ is induced during regression of a hormone-sensitive breast carcinoma cell line (Kyprianou *et al.*, 1991*b*), but it is difficult from experiments of this type to be certain that the new transcripts are integral to apoptosis as opposed to other (perhaps abortive) stress responses.

Subtractive hybridisation has also been used to identify genes whose transcription is uniquely associated with apoptosis. Several candidates are now known. Regressing secretory epithelia in mammary and prostatic glands transcribe a gene coding for a highly sulphated cell surface glycoprotein with complement-inhibitory properties (TRPM-2) (Monpetit *et al.*, 1986; Buttyan *et al.*, 1989), and several new transcripts appear in apoptotic lymphocytes (Owens *et al.*, 1991). The roles of all of these interesting new molecules have still to be established.

*Regulatory genes* influence cellular susceptibility to enter apoptosis. Some of these have been identified and are already familiar as oncogenes and oncosuppressor genes. At present, there are convincing data relating to c-*myc*, *bcl*-2, p53 and *ras*.

Expression of c-*myc* is of particular interest, as it seems to determine either continuous proliferation or apoptosis, depending on the availability of critical growth factors (Evan *et al.*, 1992). Cultured immortalised fibroblasts respond to the addition of serum growth factors by entering the proliferation cycle, and this is preceded by and apparently dependent upon transcription of c-*myc*. On withdrawal of serum growth factors, c-*myc* is down-regulated and the cells revert to a growth-arrested state in which they may remain viable for many weeks. In cells modified to express c-*myc* constitutively, however, the absence of serum growth factors is not accompanied by growth arrest. Instead, the cells remain in cycle – and some may successfully complete DNA and cellular replication – whilst substantial numbers die by apoptosis. This effect of serum starvation can be produced by other interventions that blockade some step in the proliferation cycle (for example leucine deprivation, thymidine-induced S-phase arrest or treatment with topo-isomerase II inhibitor (Bertrand *et al.*, 1991; Fanidi *et al.*, 1992)). Thus c-*myc* expression and the availability of critical growth factors (of which IGF-1 appears to be the most significant in fibroblasts) delineate three extreme cell states – *growth arrest* (c-*myc* off, growth factors absent), *population expansion* (c-*myc* on, growth factors present), and *apoptosis* (c-*myc* on, growth factors off). Study of cultured cells suggests the existence of an intermediate *high turnover state* in which susceptibility to apoptosis is high despite the fact that some cells may be competent to enter the proliferation cycle (Dive & Wyllie, 1992). Quantitative as well as qualitative consideration may determine cell transitions between these states.

Some cellular proto-oncogenes appear to 'rescue' cells from the high turnover state into the population expansion state. Amongst these is *bcl*-2 (Hockenberry *et al.*, 1990; Nunez *et al.*, 1990; Bissonnette *et al.*, 1992; Fanidi *et al.*, 1992). An elegant demonstration of this is the resistance to apoptosis of cortical thymocytes in which *bcl*-2 is engineered for constitutive expression (Sentman *et al.*, 1991; Strasser *et al.*, 1991). Such cells fail to undergo apoptosis in response to a wide variety of stimuli to which normal thymocytes are sensitive. Activation of *bcl*-2 is not unique in rescuing cells from apoptosis. High expression of the mutated *ras* oncogenes (Wyllie *et al.*, 1987) and exposure to many growth factors can have the same effect (Williams *et al.*, 1990; Koury & Bondurant, 1990) perhaps in a cell type dependent way.

Expression of wild type p53 appears to have the opposite effect, inducing apoptosis in susceptible cells (Yonish-Rouach *et al.*, 1991). Wild-type p53 is known to cause temporary G1 arrest in some cell types (Kastan *et al.*, 1991) and it is thus possible that this arrest co-operates with endogenous c-*myc* expression to initiate apoptosis.

It is not clear how these regulatory genes influence susceptibility to apoptosis. One obvious mechanisms would be the induction or depletion of the effector proteins discussed earlier. In support of this, endogenous endonuclease activity is readily demonstrated in the nuclei of fibroblasts that constitutively express c-*myc*, but is absent from cells expressing mutated Ha-*ras* at high level (Arends, M.J.: unpublished observations). It is convenient to designate as *primed* those cells in which synthesis of the effector proteins of apoptosis has been induced, and so to distinguish them both from the unprimed cells that lack effectors and cannot immediately undergo apoptosis, and from cells in the process of entering apoptosis through activation of the effectors, which we call *triggered* (Arends & Wyllie, 1991). The concept that genes regulate dynamic transitions between suseptibility and resistance to apoptosis has important implications for our understanding of regulation of normal tissue kinetics and several aspects of the behaviour of tumour cells.

Thus, during affinity maturation of the immune response, virgin circulating B cells enter the follicle centre and are stimulated by local factors to rapid cell proliferation. However, the majority of these cells are destined for almost immediate death by apoptosis: they are in the high turnover state described previously complete with high c-*myc* expression. Only a small sub-population is selected for long-term survival (and B-cell memory) on the basis of the affinity of their surface immunoglobulin for the antigen, presented on the surface of dendritic cells in the follicle centre (Liu *et al.*, 1989). This 'rescue' is associated with induction of *bcl*-2 in the surviving cells (Nunez *et al.*, 1991; Hockenberry *et al.*, 1991).

Although much less is known of the gene expression involved, an analogous situation exists in development of the nervous system. Motor neurones are generated in excess of eventual requirement, and deleted by apoptosis if their axons fail to make contact with muscle endplates, the source of growth factors which presumably rescue them from death. Rather similar processes appear to take place in the central nervous system in development of glia and the establishment of interneuronal networks in the central nervous system (Martin *et al.*, 1988; Raff, 1992).

One corollary of the hypotheses outlined above is that cells in the high turnover state include many that are primed for apoptosis and therefore susceptible to die by this means in response to a variety of lethal stimuli. Cells of the lymphoid follicle centre phenotype and at the base of intestinal crypts (another high-turnover, c-*myc*-expressing, *bcl*-2 expressing population) (Liu *et al.*, 1989; Nunez *et al.*, 1991) are indeed susceptible to apoptosis engendered by agents with widely divergent modes of action (including many used in cancer chemotherapy) (Ijiri & Potten, 1987). There are obvious implications for toxicology and cancer chemotherapy if cell sensitivity to apoptosis can be modified by manipulation of a set of regulatory genes.

Similarly, there are implications in carcinogenesis. The genes discussed above as influencing apoptosis are all familiar because of their abnormal regulation in neoplasms. One important element in the early phases of carcinogenesis may be expansion of a target population of cells capable of subsequent genetic progression to malignancy, perhaps as a result of further genetic events. This target population may normally be kept small through apoptosis. Inappropriate rescuing stimuli may therefore be oncogenic simply because they increase the number of cells available for mutagenesis. There are several potential examples, such as *ras* expression in premalignant hyperplasias of breast (Going *et al.*, 1992) and in colorectal adenomas (Williams *et al.*, 1985) and the constitutive *bcl*-2 expression of follicular lymphomas and the LMP-1-induced *bcl*-2 expression of drug-resistant Burkitt's lymphoma cell lines (Clark *et al.*, 1992; Gregory *et al.*, 1991; Henderson *et al.*, 1991).

The pervasiveness of p53 mutation in human malignancies (Levine *et al.*, 1991) may be accounted for in a similar way.

### References

ARENDS, M.J., MORRIS, R.J. & WYLLIE, A.H. (1990). Apoptosis: the role of the endonuclease. *Am. J. Pathol.*, **136**, 593-608.

ARENDS, M.J. & WYLLIE, A.H. (1991). Apoptosis: mechanisms and roles in pathology. *Int. Rev. Exp. Path.*, **32**, 223-254.

BERTRAND, R., SARANG, M., JENKIN, J., KERRIGAN, D. & POMMIER, Y. (1991). Differential induction of secondary DNA fragmentation by topo-isomerase II inhibitors in human tumor cell lines with amplified c-*myc* expression. *Cancer Res.*, **51**, 6280-6285.

BISSONNETTE, R.P., ECHEVERRI, F., MAHBOUBI, A. & GREEN, D.R. (1992). Apoptotic cell death induced by c-*myc* is inhibited by *bcl*-2. *Nature*, **359**, 552-554.

BUTTYAN, R., OLSSON, C.A., PINTAR, J., CHANG, C., BANDYK, M., NG, P.Y. & SAWCZUK, I.S. (1989). Induction of the *TRPM-2* gene in cells undergoing programmed cell death. *Mole. & Cell. Biol.*, **9**, 3473-3481.

BUTTYAN, R., ZAKERI, Z., LOCKSHIN, R. & WOGLEMUTH, D. (1988). Cascade induction of *c-fos*, *c-myc* and heat shock protein 70K transcripts during regression of rat ventral prostate gland. *Mole. Endocrinol.*, **11**, 650-656.

CLARK, H.M., JONES, D.B. & WRIGHT, D.H. (1992). Cytogenetic and molecular studies of t(14;18) in nodal and extranodal B-cell lymphoma. *J. Pathol.*, **166**, 129-137.

DIVE, C. & WYLLIE, A.H. (1992). Apoptosis and cancer chemotherapy. In Hickman, J.A. & Tritton, T.T. (eds) *Frontiers in Pharmacology: Cancer Chemotherapy*. Blackwell Scientific: Oxford (in press).

DUVALL, E., WYLLIE, A.H. & MORRIS, R.G. (1985). Macrophage recognition of cells undergoing programmed cell death. *Immunology*, **56**, 351-358.

DYSON, J.E.D., SIMMONS, D.M., DANIEL, J., MCLAUGHLIN, J.M., QUIRKE, P. & BIRD, C.C. (1986). Kinetic and physical studies of cell death induced by chemotherapeutic agents or hyperthermia. *Cell & Tissue Kinetics*, **19**, 311-324.

EASTMAN, A. (1990). Activation of programmed cell death by anticancer agents: cisplatin as a model system. *Cancer Cells*, **2**, 275-280.

EVAN, G.I., WYLLIE, A.H., GILBERT, C.S., LITTLEWOOD, T.D., LAND, H., BROOKS, M., WATERS, C.M., PENN, L.Z. & HANCOCK, D.C. (1992). Induction of apoptosis in fibroblasts by c-*myc* protein. *Cell*, **69**, 119-129.

FANIDI, A., HARRINGTON, E.A. & EVAN, G.I. (1992). Cooperative interaction between c-*myc* and *bcl*-2 proto-oncogenes. *Nature*, **359**, 554-556.

FESUS, L., DAVIES, P.J.A. & PIACENTINI, M. (1991). Molecular mechanisms in the program of cell death by apoptosis. *Eur. J. Cell Biol.*, **56**, 170-177.

FESUS, L., THOMAZY, V., AUTUORI, F., CERU, M.P., TARCSA, E. & PIACENTINI, M. (1989). Apoptotic hepatocytes become insoluble in detergents and chaotropic agents as a result of transglutaminase action. *FEBS Lett.*, **245**, 150-154.

GOING, J.J., ANDERSON, T.J. & WYLLIE, A.H. (1992). *Ras* p21 in breast tissue: association with pathology and cellular localisation. *Br. J. Cancer*, **65**, 45-50.

GREGORY, C.D., DIVE, C., HENDERSON, S., SMITH, C.A., WILLIAMS, G.T., GORDON, J. & RICKINSON, A.B. (1991). Activation of Epstein-Barr virus latent genes protects human B cells from death by apoptosis. *Nature*, **349**, 612-614.

HENDERSON, S., ROWE, M., GREGORY, C.D., CROOM-CARTER, D., WANG, F., LONGNECKER, R., KIEFF, E. & RICKINSON, A.B. (1991). Induction of *bcl-2* expression by Epstein Barr virus latent membrane protein 1 protects infected B cells from programmed cell death. *Cell*, **65**, 1107-1115.

HOCKENBERRY, D., NUNEZ, G., SCHREIBER, R.D. & KORSMEYER, S.J. (1990). *Bcl-2* is an inner mitochondrial membrane protein that blocks programmed cell death. *Nature*, **348**, 334-336.

HOCKENBERRY, D.M., ZUTTER, M., HICKEY, W., NAHM, M. & KORSMEYER, S.J. (1991). *Bcl-2* protein is topographically restricted in tissues characterized by apoptotic cell death. *Proc. Natl Acad. Sci. USA*, **88**, 6961-6965.

IJIRI, K. & POTTEN, C.S. (1987). Further studies on the response of intestinal crypt cells of different hiearchical status to eighteen different cytotoxic agents. *Br. J. Cancer*, **55**, 113-123.

ITOH, N., YONEHARA, S., ISHII, A., YONEHARA, M., MIZUSHIMA, S.-I., SAMESHIMA, M., ITASE, A., YOSHIYUKI, S. & NEGATA, S. (1991). The polypeptide encoded by the cDNA of human cell surface antigen *fas* can modulate apoptosis. *Cell*, **66**, 233-243.

KASTAN, M.B., OHYEKWERE, O., SIDRANSKY, D., VOGELSTEIN, B. & CRAIG, R.W. (1991). Participation of p53 protein in the cellular response to DNA damage. *Cancer Res.*, **51**, 6304-6311.

KERR, J.F.R., SEARLE, J., HARMON, B.V. & BISHOP, C.J. (1987). Apoptosis. In *Perspectives on Mammalian Cell Death*. Potten, C.S. (ed.) Oxford Science Publications: Oxford, p. 93-128.

KERR, J.F.K., WYLLIE, A.H. & CURRIE, A.H. (1972). Apoptosis, a basic biological phenomenon with wider implications in tissue kinetics. *Br. J. Cancer*, **26**, 239-245.

KOURY, M.J. & BONDURANT, M.C. (1990). Erythropoietin retards DNA breakdown and prevents programmed cell death in erythroid progenitor cells. *Science*, **248**, 378-381.

KYPRIANOU, N., ALEXANDER, R.B. & ISAACS, J.T. (1991*a*). Activation of programmed cell death by recombinant human tumour necrosis factor plus topoisomerase II-targeted drugs in L929 tumor cells. *J. Natl Canc. Inst.*, **83**, 346-350.

KYPRIANOU, N., ENGLISH, H.F., DAVIDSON, N.E. & ISAACS, J.T. (1991*b*). Programmed cell death during regression of the MCF-7 human breast cancer following estrogen ablation. *Cancer Res.*, **51**, 162-166.

KYPRIANOU, N., ENGLISH, H.F. & ISAACS, J.T. (1990). Programmed cell death during regression of PC-82 human prostate cancer following androgen ablation. *Cancer Res.*, **50**, 3748-3753.

LASTER, S.M., WOOD, J.G. & GOODING, L.R. (1988). Tumour necrosis factor can induce both apoptotic and necrotic forms of cell lysis. *J. Immunol.*, **141**, 2629-2634.

LENNON, S.V., MARTIN, S.J. & COTTER, T.G. (1990). Induction of apoptosis (programmed cell death) in tumour cell lines by widely diverging stimuli. *Biochem. Soc. Trans.*, **18**, 343.

LEVINE, A.J., MOMARD, J. & FINLAY, C.A. (1991). The p53 tumour suppressor gene. *Nature*, **351**, 453-456.

LIU, Y.J., JOSHUA, D.E., WILLIAMS, G.T., SMITH, C.A., GORDON, J. & MCCLENNAN, I.C.M. (1989). The mechanisms of antigen-driven selection in germinal centres. *Nature*, **342**, 929-931.

MCCONKEY, D.J., NICOTERA, P., HARTZELL, P., BELLOMO, G., WYLLIE, A.H. & ORRENIUS, S. (1989*a*). Glucocorticoids activate a suicide process in thymocytes through an elevation of cytosolic calcium concentration. *Arch. Biochem. & Biophys.*, **269**, 365-370.

MARTIKAINEN, P., KYPRIANOU, N., TUCKER, R.W. & ISAACS, J.T. (1991). Programmed cell death of nonproliferating androgen-independent prostatic cancer cells. *Cancer Res.*, **51**, 4693-4700.

MARTIN, D.P., SCHMIDT, R.E., DISTEFANO, P.S., LOWRY, O.H., CARTER, J.G. & JOHNSON, E.M. (1988). Inhibitors of protein synthesis and RNA synthesis prevent neuronal death caused by nerve growth factor deprivation. *J. Cell Biol.*, **106**, 829-844.

MONPETIT, M.L., LAWLESS, K.R. & TENNISWOOD, M. (1986). Androgen-repressed messages in the rat ventral prostate. *The Prostate*, **8**, 25-30.

MOORE, J.V. (1987). Death of cells and necrosis of tumours. In Potten, C.S. (ed.) *Perspectives in Mammalian Cell Death*. Oxford: Oxford University Press, p 295-325.

NUNEZ, G., HOCKENBERRY, D., MCDONNELL, T.J., SORENSEN, C.M. & KORSMEYER, S.J. (1991). Bcl-2 maintains B cell memory. *Nature*, **353**, 71-73.

NUNEZ, G., LANDON, L., HOCKENBERRY, D., ALEXANDER, M., MCKEARN, J.P. & KORSMEYER, S.J. (1990). *J. Immunol.*, **144**, 3602.

OWENS, G.P., HAHN, W. & COHEN, J.J. (1991). Identification of mRNAs associated with programmed cell death in immature thymocytes. *Mol. & Cell. Biol.*, **11**, 4177-4188.

PIACENTINI, M., AUTOUORI, F., DINI, L., FARRAEE, M.G., GHIBELLI, L., PIREDDA, L. & FESUS, L. (1991). Tissue transglutaminase is specifically expressed in neonatal rat liver cells undergoing apoptosis upon epidermal growth factor stimulation. *Cell Tissue Res.*, **263**, 227–235.

RAFF, M. (1992). Solid controls on cell survival and cell death. *Nature*, **356**, 397–400.

SARRAF, C.E. & BOWEN, I.D. (1988). Proportions of mitotic and apoptotic cells in a range of untreated experimental tumours. *Cell & Tissue & Kinetics*, **21**, 45–49.

SAVILL, J.S., DRANSFIELD, I., HOGG, N. & HASLETT, C. (1990). Vitronectin receptor-mediated phagocytosis of cells undergoing apoptosis. *Nature*, **343**, 170–173.

SAVILL, J.S., WYLLIE, A.H., HENSON, J.E., WALPORT, M.J.J., HENSON, P.M. & HASLETT, C. (1989). Macrophage phagocytosis of aging neutrophils inflammation. Programmed cell death in the neutrophil leads to recognition by macrophage. *J. Clin. Invest.*, **83**, 865–875.

SEARLE, J., LAWSON, T.A., ABBOTT, P.J., HARMON, B.V. & KERR, J.F.R. (1975). An electorn microscopy study of the mode of cell death induced by cancer-chemotherapeutic agents in populations of proliferating normal and neoplastic cells. *J. Pathol.*, **116**, 129–138.

SENTMAN, C.L., SHUTTER, J.R., HOCKENBERG, D., KANAGAWA, D. & KORSMEYER, S.J. (1991). *bcl-2* inhibits multiple forms of apoptosis but not negative selection in thymocytes. *Cell*, **67**, 879–888.

SMITH, C.A., WILLIAMS, G.T., KINGSTON, R., JENKINSON, E.J. & OWEN, J.J.T. (1989). Antibodies to CD3/T receptor complex induce cell death by apoptosis in immature thymic cultures. *Nature*, **337**, 181–184.

STRASSER, A., HARRIS, A.W. & CORY, S. (1991). *bcl*-2 transgene inhibits T cell death and perturbs thymic self-censorship. *Cell*, **67**, 889–899.

SZENDE, B., ZALATINI, A. & SCHALLY, A.W. (1989). Programmed cell death (apoptosis) in pancreatic cancers of hamsters after treatment with analogs of both luteinizing hormone-releasing hormone and somatostatin. *Proc. Natl Acad. Sci.*, **86**, 1643–1647.

TRAUTH, B.C., KLAS, C., PETERS, A.M., MATZKU, S., MOLLER, P., FALK, W., DEBATIN, K.M. & KRAMER, P.H. (1989). Monoclonal antibody-mediated tumour regresssion by induction of apoptosis. *Science*, **245**, 301–305.

WILCOCK, C. & HICKMAN, J.A. (1988). Characterisation of a $Na^+$/$K^+$/$Cl^-$ cotransporter in alkylating agent-sensitive L1210 murine leukemia cells. *Biochim. Biophys. Acta*, **946**, 359–367.

WILLIAMS, A.R.W., PIRIS, J., SPANDIDOAS, D.A. & WYLLIE, A.H. (1985). Immunohistochemical detection of the *ras* oncogene p21 product in an experimental tumour and in human colorectal neoplasms. *Br. J. Cancer*, **52**, 687–693.

WILLIAMS, G.T., SMITH, C.A., SPOONCER, E., DEXTER, T.M. & TAYLOR, D.R. (1990). Haemopoietic colony stimulating factors promote cell survival by suppressing apoptosis. *Nature*, **343**, 76–78.

WYLLIE, A.H. (1980). Glucocorticoid-induced thymocyte apoptosis is associated with endogenous endonuclease activation. *Nature*, **284**, 555–556.

WYLLIE, A.H., KERR, J.F.R. & CURRIE, A.R. (1980). Cell death: the significance of apoptosis. *Intern. Rev. Cytol.*, **68**, 251–306.

WYLLIE, A.H. & MORRIS, R.G. (1982). Hormone-induced cell death. Purification and properties of thymocytes undergoing apoptosis after glucocorticoid treatment. *Amer. J. Pathol.*, **109**, 78–87.

WYLLIE, A.H., MORRIS, R.G., SMITH, A.L. & DUNLOP, D. (1984). Chromatin cleavage in apoptosis: association with condensed chromatin morphology and dependence on macromolecular synthesis. *J. Pathol.*, **142**, 67–77.

WYLLIE, A.H., ROSE, K.A., MORRIS, R.G., STEEL, C.M., FOSTER, E. & SPANDIDOS, D.A. (1987). Rodent fibroblast tumours expressing *myc* and *ras* genes. Growth, metastasis and endogenous oncogene expression. *Br. J. Cancer*, **56**, 251–259.

YONISH-ROUACH, E., RESNITSKY, D., LOTEM, J., SACHS, K., KIMCHI, A. & OREN, M. (1991). Wild-type *p53* induces apoptosis of myeloid leukaemic cells that is inhibited by interleukin-6. *Nature*, **352**, 345–347.